# Cytosine-Rich Oligonucleotide and Electrochemically Reduced Graphene Oxide Nanocomposite for Ultrasensitive Electrochemical Ag^+^ Sensing

**DOI:** 10.3390/nano14090775

**Published:** 2024-04-29

**Authors:** Nasir Abbas, Seung Joo Jang, Tae Hyun Kim

**Affiliations:** Department of Chemistry, Soonchunhyang University, Asan 31538, Republic of Korea; nasir@sch.ac.kr (N.A.); seen813@sch.ac.kr (S.J.J.)

**Keywords:** reduced graphene oxide, DNA hairpin, silver ion, electrochemical aptasensor

## Abstract

Silver ions (Ag^+^) are crucial in various fields, but pose environmental and health risks at high concentrations. This study presents a straightforward approach for the ultra-trace detection of Ag^+^, utilizing a composite of a cytosine-rich oligonucleotide (CRO) and an electrochemically reduced graphene oxide (ERGO). Initially, ERGO was synthesized on a glassy carbon electrode (GCE) through the reduction of graphene oxide (GO) via cyclic voltammetry. A methylene blue-tagged CRO (MB-CRO) was then anchored to the ERGO surface through π–π interactions, resulting in the formation of an MB-CRO-modified ERGO electrode (MB-CRO/ERGO-GCE). The interaction with Ag^+^ ions induced the formation of silver-mediated C-Ag^+^-C coordination, prompting the MB-CRO to adopt a hairpin structure. This conformational change led to the desorption of the MB-CRO from the ERGO-GCE, causing a variation in the redox current of the methylene blue associated with the MB-CRO. Electrochemical assays revealed that the sensor exhibits extraordinary sensitivity to Ag+ ions, with a linear detection range from 1 femtomolar (fM) to 100 nanomolars (nM) and a detection limit of 0.83 fM. Moreover, the sensor demonstrated high selectivity for Ag^+^ ions and several other benefits, including stability, reproducibility, and straightforward fabrication and operational procedures. Additionally, real sample analyses were performed using the modified electrode to detect Ag^+^ in tap and pond water samples, yielding satisfactory recovery rates.

## 1. Introduction

Silver ion (Ag^+^) is a precious metal ion valued for its rarity and applications in biomedicine [1], electronics [2], and various other fields [3,4,5,6]. At low concentrations, Ag^+^ exhibits beneficial properties such as anti-inflammatory [7], sterilizing [8], deodorizing [9], and wound-healing effects [10], and it is also known to inhibit certain bacteria [11], fungi, and viruses [12,13]. However, Ag^+^ can also pose environmental and health risks [14,15]. Silver is often found as an impurity in ores and can enter the environment through industrial waste [16,17,18]. Additionally, the widespread use of silver in industrial processes, including chemistry [19,20], medicine [21], photography [22], and electronics [23], leads to its discharge into the environment through industrial waste, often contaminating water and soil [24]. This environmental infiltration poses significant risks to plant growth [15] and ecological balance. Accumulation of Ag^+^ in animals and plants, coupled with its potential entry into the human body through the food chain, presents health hazards such as elevated urine silver excretion, argyria (a skin condition), cardiac enlargement, growth retardation, and liver damage [1,25,26]. Thus, the U.S Environmental Protection Agency (USEPA) and the World Health Organization (WHO) have set the toxic concentration standard for Ag^+^ at 1.6 nM. Additionally, the maximum permissible concentration of Ag^+^ in drinking water is specified as 0.93 μM and 0.36 μM, respectively. Furthermore, WHO limited the threshold for exposure of silver in water for fish and microorganisms to lower than 1.6 nmol L^−1^ [27,28]. Consequently, the detection of trace amounts of Ag^+^ is crucial for environmental protection and public health [29,30,31,32,33]. 

Conventional methods like atomic absorption spectrometry (AAS) [34,35,36], fluorescence [37,38], colorimetric [39], and inductively coupled plasma mass spectrometry (ICP-MS) offer high accuracy for Ag^+^ detection [40,41], but are often expensive and require complex instrumentation. These techniques typically require specialized equipment, high sample volumes, and lengthy processing times. While researchers have developed sensors for Ag^+^ detection using various spectroscopic techniques, these often involve complex design and synthesis procedures. 

In recent years, electrochemical analysis technology has gained widespread adoption for detecting heavy metal ions [4,28], biomolecules [42], and food additives due to its high sensitivity and cost-effectiveness [43,44,45]. Consequently, numerous researchers have studied graphene composites to enhance sensor performance. Among these composites, reduced graphene oxide (RGO) has been considered as a promising material for improving the performance of electrochemical biosensors. RGO offers a large surface area, good biocompatibility, and excellent electrocatalytic activity, which are attributed to its abundant edge-plane-like defects and large surface area, facilitating rapid heterogeneous electron transfer [46,47,48,49]. Furthermore, RGO can readily bind with single-stranded DNA through π–π stacking. DNA aptamer-based electrochemical biosensors have emerged as particularly promising due to their advantages: enhanced sensitivity [50], rapid analysis [51], cost-effectiveness [52], straightforward fabrication [53], miniaturization potential [52], and suitability for on-site detection [54,55,56]. 

**Scheme 1:**
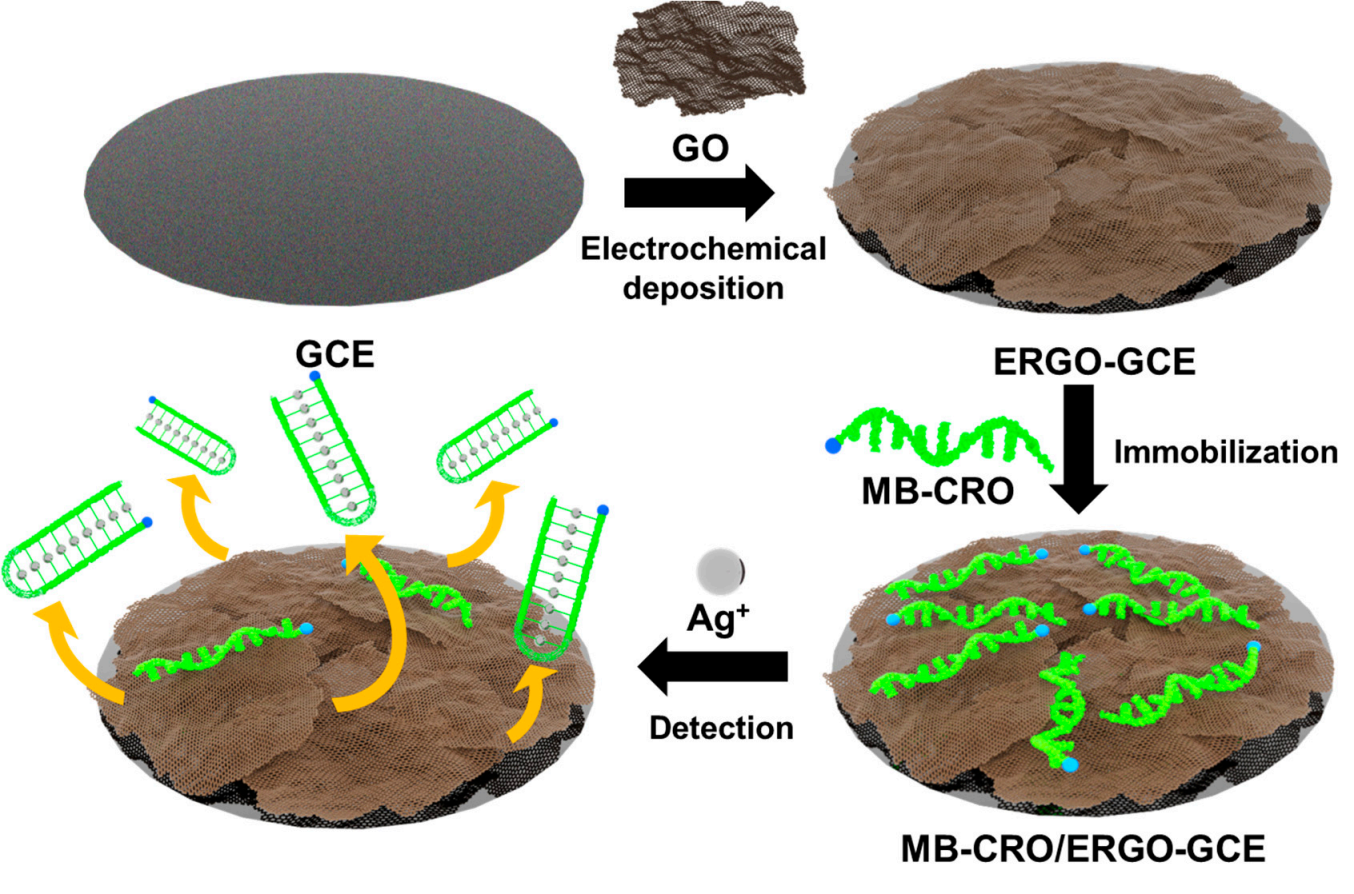
Fabrication and Ag^+^ detection mechanism of the MB-CRO/ERGO-GCE aptasensor.

Previously, Prof. Han’s group suggested that aptasensors detect silver ions via probe packing density [57]. Their approach involved the use of cytosine-rich oligonucleotides (CRO) tagged with methylene blue, which were self-assembled onto the gold electrode surface via thiol groups from the aptamer’s 5′-terminal. In contrast, we propose methylene blue (MB)-tagged CRO modified on the electrochemically reduced graphene oxide (ERGO) deposition glassy carbon electrode (GCE) surface; this method does not require additional preparation of DNA such as thiolate. Additionally, single-stranded DNA can be reversibly absorbed simply by dipping the electrode in a DNA solution. To elaborate, Scheme 1 illustrates the fabrication process of the sensor and its Ag^+^ detection mechanism, demonstrating its simplicity, speed, and effectiveness. Briefly, ERGO is prepared and deposited onto a glassy carbon electrode through the direct reduction of GO using cyclic voltammetry (CV), following established protocols [58,59]. Subsequently, MB-CRO is immobilized onto the ERGO-modified GCE (ERGO-GCE) via π–π stacking [60,61]. The introduction of Ag^+^ prompts the MB-CRO on the ERGO-GCE, leading it to adopt a hairpin structure facilitated by a specific C-Ag^+^-C coordination [62,63,64]. This structural shift triggers the detachment of the MB-CRO from the ERGO surface, resulting in a marked alteration in the electrochemical signal of the MB tag. The sensor is characterized by its straightforward fabrication, ease of analysis, ultra-sensitivity, and high selectivity, making it a practical tool for Ag^+^ ion detection.

## 2. Materials and Methods

### 2.1. Materials

The cytosine-rich oligonucleotide aptamer (MB-CRO), with the sequence 5′-Methylene Blue-CCCCCCCCCCCCCCCCCCCCCCCC-3′ (24C) [65], was sourced from BIONEER Corporation (Daejeon, Republic of Korea). Graphite powder, phosphate buffer saline (PBS), Tris(hydroxymethyl)aminomethane hydrochloride (Tris-HCl), and potassium hexacyanoferrate (III) (K_3_[Fe(CN)_6_]) were procured from Sigma-Aldrich, Inc. (St. Louis, MO, USA). All chemicals employed were of analytical grade and utilized as received, without any further purification. The aqueous solutions were prepared using deionized water with a resistivity greater than 18 MΩcm, produced by a Millipore water purification system (MilliQ, Millipore Korea, Co., Ltd., Seoul, Republic of Korea).

### 2.2. Electrochemical Measurements

Electrochemical measurements were performed using a Model 660D electrochemical workstation (CH Instruments, Austin, TX, USA) with a conventional three-electrode cell at room temperature (25 °C). GCE, ERGO-GCE, and MB-CRO/ERGO-GCE served as working electrodes, with Ag/AgCl (3 M NaCl) as the reference electrode and a Pt wire as the counter electrode. All measurements, including cyclic voltammetry (CV), differential pulse voltammetry (DPV), and electrochemical impedance spectroscopy (EIS), were conducted in 10 mM phosphate-buffered saline (PBS) or 7.4 pH Tris buffer solution. CV experiments employed a potential scan from −0.2 to 0.6 V (vs. Ag/AgCl) at 10 mV/s with a 1 mV sampling interval. DPV utilized a scan range of 0.1 to −0.6 V (vs. Ag/AgCl), a pulse amplitude of 0.04 V, a pulse width of 0.2 s, and a pulse time of 0.5 s. EIS measurements were carried out in 10 mM Tris solution containing 5 mM [Fe(CN)_6_]^4−^ at the formal potential of 0.222 V (vs. Ag/AgCl), with an AC voltage amplitude of 10 mV and a frequency range of 106 to 10^−1^ Hz. ZSimpWin 3.21 software (AMETEK. Inc., Oak Ridge, TN, USA) was used for EIS data fitting.

### 2.3. Fabrication of MB-CRO/ERGO-GCE

GO was produced via a modified Hummers method [66]. The resultant GO was ultrasonicated in 10 mM PBS buffer (pH 7.4) for 1 h, yielding a uniform yellow-brown solution (0.3 mg/mL in 10 mM PBS buffer, pH 7.4). Before constructing the ERGO-GCE, the GCE was polished to a mirror finish with alumina powders of 0.1, 0.3, and 0.05 µm, respectively, sonicated for 10 min in water and ethanol solution, and then rinsed with deionized water. The ERGO-GCE was fabricated by applying CV from 0.8 to −1.5 V vs. Ag/AgCl at a scan rate of 10 mV/s for three cycles in the GO solution (0.3 mg/mL in 10 mM PBS buffer, pH 7.4), using GCE as the working electrode. Finally, MB-CRO was immobilized onto the ERGO-GCE. The ERGO/GCE was incubated in 1 μM MB-CRO solution (10 mM Tris buffer, pH 7.4) for varying (10, 20, 30, 40, 50, 60, 70, and 80 min) incubation times. After incubation, the MB-CRO/ERGO-GCE was washed with buffer solution and dried at room temperature before use.

## 3. Results and Discussion

**Figure 1:**
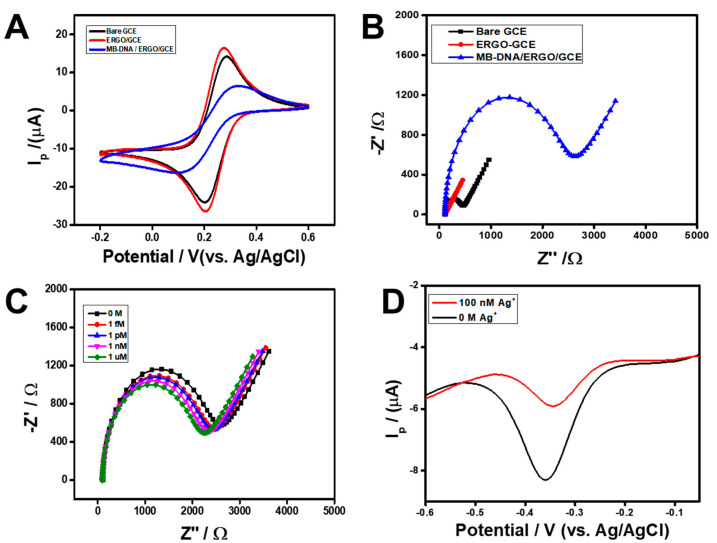
(**A**) CV curves for GCE, ERGO−GCE, and MB−CRO/ERGO−GCE obtained at a scan rate of 10 mV/s in 0.1 M KCl containing 10 mM [Fe(CN)_6_]^3−^. (**B**) Nyquist plots representing the electrochemical impedance of GCE, ERGO−GCE, and MB−CRO/ERGO−GCE in 10 mM Tris buffer with 10 mM [Fe(CN)_6_]^3−^. (**C**) EIS Nyquist plots of MB−CRO/ERGO−GCE upon exposure to varying Ag^+^ concentrations (0 M, 1 fM, 1 pM, 1 nM, and 1 µM) in 10 mM Tris buffer. (**D**) Differential pulse voltammograms (DPV) of MB−CRO/ERGO−GCE in the absence and presence of 100 nM Ag^+^ in 10 mM Tris buffer (pH 7.4).

The fabrication process of MB-CRO/ERGO-GCE was characterized using CV and electrochemical impedance spectroscopy (EIS), with [Fe(CN)_6_]^3−^ as a redox probe. In 0.1 M KCl containing 10 mM K_3_[Fe(CN)_6_], CV revealed a well-defined redox peak pair on the bare GCE (Figure 1A) with a peak-to-peak separation (ΔE_p_) of 84 mV. Modification with ERGO significantly increased the peak current (I_p_) and decreased ΔE_p_ (70 mV), indicating enhanced electron transfer due to ERGO’s conductivity. Immobilization of MB-CRO on ERGO-GCE resulted in a substantial decrease in I_p_ and a noticeable increase in ΔE_p_ (205 mV). This suggests that the negatively charged MB-CRO acts as an insulator, thereby impeding electron transfer. These observations confirm the successful construction of the MB-CRO/ERGO-GCE. EIS further validated the modification process (Figure 1B). The Nyquist plot shows the charge transfer resistance (R_ct_) represented by the semicircle diameter at high frequencies. ERGO modification on the GCE resulted in a reduction in the R_ct_ value from 206 Ω (bare GCE) to 189.2 Ω (ERGO-GCE), attributed to the catalytic effect of ERGO, which facilitates rapid electron transfer. Conversely, the attachment of MB-CRO to the ERGO-GCE surface caused a significant rise in R_ct_ to 2156 Ω, attributable to the DNA structure’s low conductivity, which restricts electron movement. This further corroborates the successful fabrication of the MB-CRO/ERGO-GCE.

Furthermore, as shown in Figure S2, we calculated the active surface areas of both bare GCE and ERGO-GCE to validate the successful deposition of ERGO onto the electrode surface. The active surface areas were determined to be 0.059 cm^2^ for bare GCE and 0.063 cm^2^ for ERGO-GCE, respectively.

The potential of MB-CRO/ERGO-GCE as an electrochemical sensor for Ag^+^ detection was demonstrated through an assessment of EIS and DPV measurements upon Ag^+^ introduction. Figure 1C shows that increasing Ag^+^ concentrations corresponded to a decrease in R_ct_, with more pronounced decreases at higher concentrations. This suggests that Ag^+^ binding to MB-CRO on the electrode surface triggers a conformational change, leading to MB-CRO detachment and a decrease in the barrier-to-electron transfer. To exploit the MB tag for signal transduction, DPV measurements were performed in 10 mM Tris buffer (pH 7.4). Figure 1D shows the DPV curves, with the MB reduction peak at −0.35 V confirming MB-CRO immobilization. Upon Ag^+^ addition, a significant decrease in the MB peak current was observed. This can be attributed to the increased distance between the MB tag and the electrode surface due to Ag^+^-induced conformational changes and subsequent MB-CRO detachment. This experimental observation provides compelling evidence supporting the sensor’s capability to detect Ag^+^ ions.

**Figure 2:**
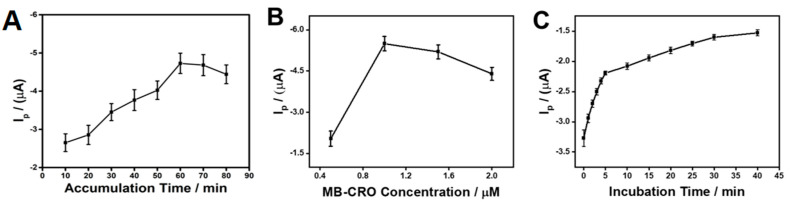
(**A**) Effect of MB-CRO immobilization time on the sensor response ([MB-CRO] = 1 μM). (**B**) Effect of MB-CRO concentration for immobilization on the sensor response (MB-CRO accumulation time = 60 min). (**C**) Effect of Ag^+^ incubation time on the sensor response ([MB-CRO] = 1 μM MB-CRO, [Ag^+^] = 1μM, MB-CRO accumulation time = 60 min).

Accumulation time for MB−CRO (1 µM) was 60 min, and incubation time for Ag^+^ detection was 5 min. To optimize the sensor’s performance, we investigated the effects of accumulation time, MB−CRO concentration for immobilization, and incubation time for the Ag^+^ sensing reaction on the voltammetric response in 10 mM Tris buffer (Figure 2). The sensor response increased with the MB−CRO immobilization time up to 60 min, indicating a gradual increase in immobilized MB−CRO (Figure 2A). Beyond 60 min, the response decreased, suggesting potential overcrowding or desorption. Therefore, 60 min was chosen as the optimal immobilization time. Similarly, the sensor response displayed a maximum at an MB−CRO concentration of 1 μM (Figure 2B). This suggests a balance between sufficient aptamer coverage and potential steric hindrance at higher concentrations. Moreover, the voltammetric response to Ag^+^ detection grew with longer incubation times, stabilizing at 5 min (Figure 2C). Therefore, a 5 min incubation period was deemed sufficient for the reaction.

**Figure 3:**
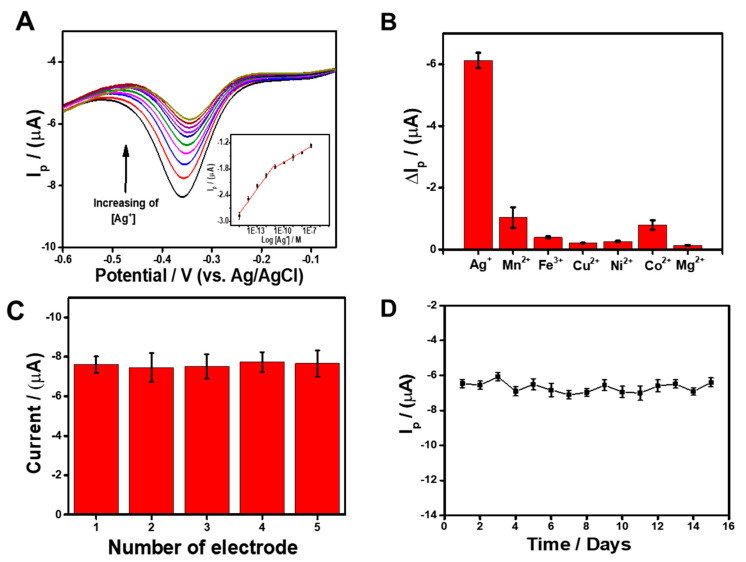
(**A**) DPV responses upon exposure to increasing Ag^+^ concentrations (1 fM–100 nM) in 10 mM Tris buffer. Inset: calibration plot showing the linear relationship between peak current (ΔI_p_) and Ag^+^ concentration (log scale). (**B**) DPV responses in the presence of 100 nM Ag^+^ and 1 μM of various potentially interfering metal ions (Mn^2+^, Fe^3+^, Cu^2+^, Ni^2+^, Co^2+^, Mg^2+^). (**C**) Reproducibility of sensor response for five independently fabricated electrodes. (**D**) Sensor stability over 15 days in 10 mM Tris buffer.

The analytical performance of the MB-CRO/ERGO-GCE sensor for Ag^+^ detection was evaluated under optimized conditions, focusing on sensitivity, selectivity, reproducibility, and stability (Figure 3). In Figure 3A, DPV curves of the MB-CRO/ERGO-GCE are shown, with varying concentrations of Ag^+^ in 10 mM Tris buffer solution (pH 7.4). As the Ag^+^ concentration increased from 1 fM to 100 nM, the sensor’s peak current values gradually decreased. The calibration plot, illustrating the relationship between Ag^+^ concentrations and I_p_, is depicted in the inset of Figure 3A. The plot reveals two distinct linear regions, each associated with different response characteristics. At lower concentrations, the sensor responded rapidly as Ag^+^ ions quickly interact with the electrode surface. At higher concentrations, the response slowed, resulting in two separate linear ranges due to this differential behavior. The first linear range, from 1 fM to 10 pM, is described by the regression equation of I_p_ (μA) = 0.26 Log[Ag^+^] + 1.2 (R^2^ = 0.98). The second linear range, from 10 pM to 100 nM, follows the regression equation of Ip (μA) = 0.12 Log[Ag^+^] − 0.44 (R^2^ = 0.99). Table 1 compares the performance of the MB-CRO/ERGO-GCE sensor with previously reported Ag^+^ sensors. The aptasensor we proposed exhibited an exceptional linear range and surpassed previously reported sensors in terms of sensitivity, achieving a remarkable limit of detection (LOD) of 0.83 fM. The sensor displayed excellent selectivity for Ag^+^ detection (Figure 3B). The peak current significantly changed only upon Ag^+^ addition (100 nM), while negligible responses were observed for other metal ions (Mn^2+^, Fe^3+^, Cu^2+^, Ni^2+^, Co^2+^, Mg^2+^) at a higher concentration (1 μM). The sensor also exhibited good reproducibility (Figure 3C). DPV responses from five independently fabricated electrodes showed minimal variation, indicating consistent sensor performance. Furthermore, the sensor demonstrated excellent long-term stability (Figure 3D). After 15 days of storage at room temperature, the peak current remained at 98.9% of its initial value, highlighting the sensor’s reliable performance.

To assess real-world applicability, the sensor’s performance was evaluated in tap water and pond water samples spiked with Ag^+^ (Table 2). Samples were prepared by diluting the real water with 10 mM Tris buffer (pH 7.4). The standard addition method was used to quantify Ag^+^. The results demonstrated good recovery rates and low relative standard deviation (RSD) values, indicating the sensor’s potential for Ag^+^ detection in real-world samples.

## 4. Conclusions

This work successfully demonstrates a novel and highly sensitive electrochemical aptasensor for Ag^+^ detection. The sensor leverages the specific interaction between MB-CRO and ERGO, achieving an ultra-high detection limit of 0.83 fM, surpassing current methods of Ag^+^ detection. Additionally, the sensor exhibits excellent selectivity for Ag^+^ ions, minimizing interference from other metal ions in real-world samples. The straightforward fabrication process and rapid analysis capabilities make the sensor readily deployable for on-site monitoring. Furthermore, the sensor demonstrates good reproducibility and stability, maintaining consistent performance across multiple fabrications and over time. The successful application of the sensor for Ag^+^ detection in real-world tap and pond water samples validates its potential for practical applications in environmental monitoring. This approach paves the way for developing highly sensitive and selective aptasensors for the detection of various environmental contaminants beyond Ag^+^. Future research will explore the sensor’s suitability for monitoring silver nanoparticle pollution and other areas of environmental and biological analysis.

## Figures and Tables

**Table 1:** Comparison of Ag^+^ sensor performance.

Materials	Technique	Linear Range	LOD	Ref.
BiOI NSs	PEC	0–300 μM	0.21 μM	[28]
SQDs/Au	DPV	0.1 nM–3 μM	71 pM	[67]
Au-IDA	SWV	0.25 nM~2 nM	106 nM	[68]
CPM-Pt	UV-vis	0.5~10 pM	1.1 pM	[29]
Oligonucleotide-Based Au Electrode	DPV	10–200 nM	10 nM	[57]
Pt@ZIF-8 nanozyme	Colorimetry	0.1–1000.0 nM	0.034 nM	[69]
MB-CRO/ERGO-GCE	DPV	1 fM~100 nM	0.83 fM	This work

**Table 2:** Detection of Ag^+^ concentrations in real samples using MB-CRO/ERGO-GCE (n = 3).

Samples	Added	Found	Recovery (%)	RSD (%)
Tap Water	100 fM	98.4 fM	98.4	3.61
	100 pM	98.6 pM	98.6	1.74
	100 nM	97.7 nM	97.7	135
Pond Water	100 fM	99.4 fM	99.4	3.15
	100 pM	97.6 pM	97.6	2.48
	100 nM	98.1 nM	98.1	1.78

## Data Availability

Data are contained within the article and Supplementary Materials.

## Supplementary Materials

The following supporting information can be downloaded at: https://www.mdpi.com/article/10.3390/nano14090775/s1, Figure S1: Raman spectra of GO and ERGO; Figure S2. CV curves at various scan rates from 0.01 to 0.1 V/s at (A) bare GCE and (C) ERGO-GCE. (B) Linear plots of υ^1/2^ vs. redox peak currents (I_pa_/I_pc_) at (B) bare GCE and (D) ERGO-GCE in 5 mM [Fe(CN)_6_]^3–^ in 0.1M KCl solution.
