# Supplementary material for: Cytosine-Rich Oligonucleotide and Electrochemically Reduced Graphene Oxide Nanocomposite for Ultrasensitive Electrochemical Ag+ Sensing
Source: Nanomaterials (Basel). 2024 Apr 29;14(9):775. doi: 10.3390/nano14090775 (PMC11085715; doi:10.3390/nano14090775)
Supplement: Supplementary file 1 [file nanomaterials-14-00775-s001.zip › nanomaterials-2966331-supplementary.pdf]

Supplementary Materials

for

Cytosine-Rich Oligonucleotide and Electrochemically Reduced Graphene Oxide Nanocomposite for Ultrasensitive Electro-chemical Ag^+ Sensing

Nasir Abbas†, Seung Joo Jang† and Tae Hyun Kim*

Department of Chemistry, Soonchunhyang University, Asan 31538, Republic of Korea

***Corresponding Author:** Tae Hyun Kim (E-mail: thkim@sch.ac.kr).

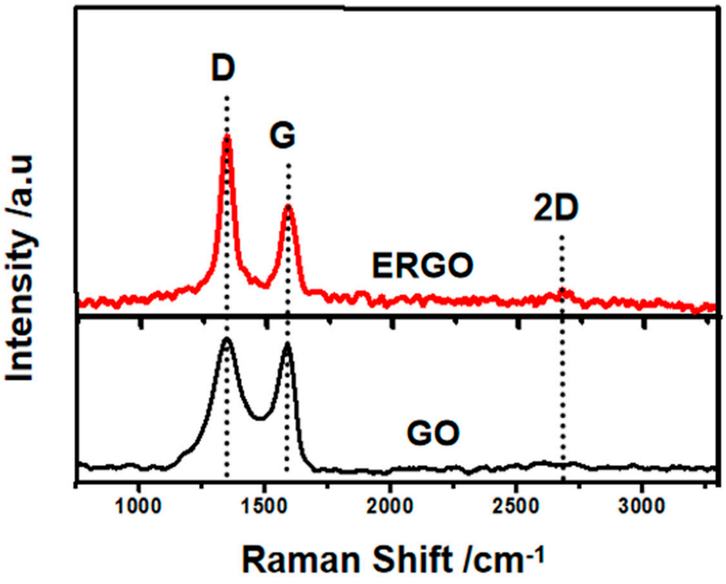

Figure S1. Raman spectra of GO and ERGO

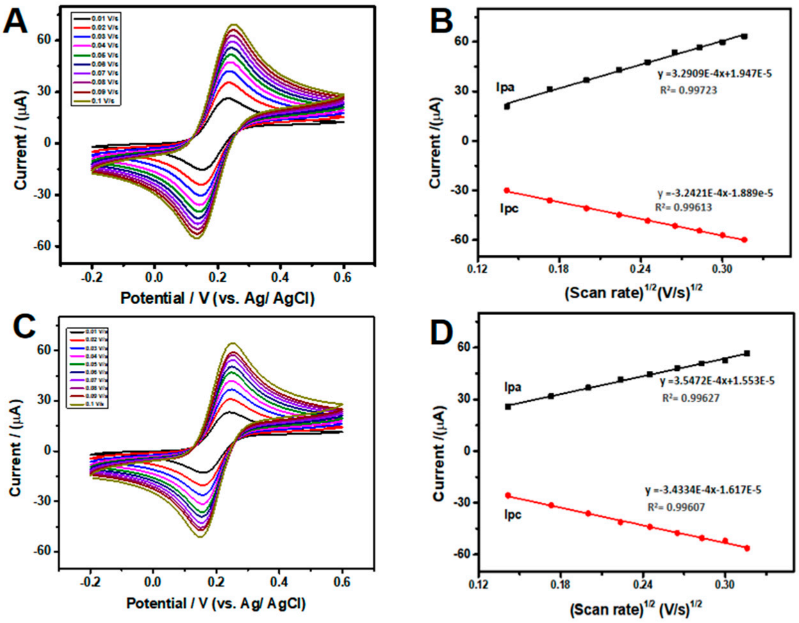

Figure S2. CV curves at various scan rates from 0.01 to 0.1 V/s at (A) bare GCE and (C) ERGO-GCE. (B) Linear plots of $\upsilon^{1/2}$ vs. redox peak currents (I_{pa}/I_{pc}) at (B) bare GCE and (D) ERGO-GCE in 5 mM $[Fe(CN)_6]^{3-}$ in 0.1M KCl solution.
